# Characterizing linkage disequilibrium and evaluating imputation power of human genomic insertion-deletion polymorphisms

**DOI:** 10.1186/gb-2012-13-2-r15

**Published:** 2012-02-29

**Authors:** James T Lu, Yi Wang, Richard A Gibbs, Fuli Yu

**Affiliations:** 1 Human Genome Sequencing Center, Baylor College of Medicine, One Baylor Plaza, Houston, Texas 77030, USA; 2 Department of Structural and Computational Biology, Baylor College of Medicine, One Baylor Plaza, Houston, Texas 77030, USA; 3 Department of Molecular and Human Genetics, Baylor College of Medicine, One Baylor Plaza, Houston, Texas 77030, USA

## Abstract

**Background:**

Indels are an important cause of human variation and central to the study of human disease. The 1000 Genomes Project Low-Coverage Pilot identified over 1.3 million indels shorter than 50 bp, of which over 890 were identified as potentially disruptive variants. Yet, despite their ubiquity, the local genomic characteristics of indels remain unexplored.

**Results:**

Herein we describe population- and minor allele frequency-based differences in linkage disequilibrium and imputation characteristics for indels included in the 1000 Genomes Project Low-Coverage Pilot for the CEU, YRI and CHB+JPT populations. Common indels were well tagged by nearby SNPs in all studied populations, and were also tagged at a similar rate to common SNPs. Both neutral and functionally deleterious common indels were imputed with greater than 95% concordance from HapMap Phase 3 and OMNI SNP sites. Further, 38 to 56% of low frequency indels were tagged by low frequency SNPs. We were able to impute heterozygous low frequency indels with over 50% concordance. Lastly, our analysis also revealed evidence of ascertainment bias. This bias prevents us from extending the applicability of our results to highly polymorphic indels that could not be identified in the Low-Coverage Pilot.

**Conclusions:**

Although further scope exists to improve the imputation of low frequency indels, our study demonstrates that there are already ample opportunities to retrospectively impute indels for prior genome-wide association studies and to incorporate indel imputation into future case/control studies.

## Background

The ubiquity of insertion-deletion polymorphism (indel) events in the human genome testifies to their relative importance in human variation [1,2]. Indels are the second most frequent polymorphism type and are thought to be more polymorphic than SNPs [3,4]. Systematic detection of indels from large population cohorts has been limited by technological challenges [5-7]. However, the advent of next generation sequencing (NGS) platforms, which affordably interrogate genomes within reasonable timeframes, presents new opportunities to detect short indels (< 50 bp) with confidence.

The 1000 Genomes Project (1000G) is the exemplary project using NGS to investigate human genetic variants on a large scale. The 1000G Low-Coverage Pilot sequenced 179 individuals to approximately 3.6X coverage by whole genome shotgun sequencing: 60 from the Centre d'Etude du Polymorphisme Humain collected in Utah, USA, with ancestry from northern and western Europe (CEU); 60 Han Chinese in Beijing, China (CHB) and Japanese in Tokyo, Japan (JPT); and 59 Yoruba in Ibadan, Nigeria (YRI). This pilot identified, on average, 361,669 short bi-allelic indel (< 50 bp) variants in each individual. Based on computation prediction, 890 indels in coding and splice site regions were classified as loss-of-function (LOF) variants [2].

Short indels in coding, promoter, and splice site regions are well known to impact protein function and cause disease. For example, indels may manifest themselves as a triplet repeat or microsatellite instability in diseases such as Huntington's disease, fragile X syndrome [8], and non-hereditary non-polyposis colorectal carcinoma [9], as in-frame deletion in cystic fibrosis [10], and as a frame-shift deletion in diseases such as Duchenne's and Becker's muscular dystrophy [11].

As a large contributor to genetic variation in human, indels are a plausible cause for some of the missing hereditability in current genome-wide association studies (GWAS) [12]. In a recent study, Mills *et al*. [1] identified over 1,102 indels in genes that were in high linkage disequilibrium (LD; r^2 ^> 0.8) with SNPs that had genome-wide significance in 118 previously published GWAS, suggesting that indels may be causative for these disease phenotypes. The prevalence and functional importance of indels in the human genome motivates investigation of the LD characteristics around indel events. LD degrades over physical distance due to mutations, genetic recombination, genetic drift and natural selection [13]. The process and strength likely differ in indel loci relative to SNPs, as some indel are in genomic repeat loci where the mutation rates are hundreds of times higher than the genome-wide average [14-16].

Characterizing LD between indels and SNPs is essential to our understanding of the landscape of genomic variation [16-20] and to improving power to discover associations with disease phenotypes. In particular, imputation methods are used to enhance variant detection power in GWAS. Imputation expands the set of variants that can be evaluated, thus allowing identification of putative variants while reducing the cost of genotyping individuals [3,21-23]. The high resolution 1000G dataset, with a large representation of low frequency variants in the population results, provides opportunities to identify new recombinant haplotypes and haplotype groupings in studied populations [2,13,24]. For this study, we utilized the 1,330,158 bi-allelic indels and 14,894,361 SNPs released by the 1000G Low-Coverage Pilot [2] and characterized the LD patterns around indels conditioned on ethnicity, minor allele frequency (MAF), length, and LOF status. We also provide to the community a look up table of indels that are in high LD (r^2 ^> 0.80) with nearby SNPs [25] for future GWAS. Using this information, we then evaluated the ability to impute indels from different SNP panels.

## Results

### Features of 1000G indel data

#### Analysis of insertions and deletions and indel MAF distribution

Indels from the 1000G Low-Coverage Pilot included a preponderance of deletions over insertions for all populations. For CEU there were 404,476 deletions to 323,599 insertions (YRI, 551,391 to 323,599; CHB+JPT, 361,339 to 305,301), a 25% higher representation. This bias partially resulted from the bias in calling methods that were based on alignment. However, the relative increase of deletions is also suggestive of a mutational process that favors slippage of primers in a forward direction rather than in reverse [26].

In examining the MAF distribution of indels detected from NGS, we found that the distribution was consistent with studies of indels detected from sequence traces [1,6]. MAF distributions for CEU and CHB+JPT indels, excluding singletons, were found to have similar proportions of low frequency (MAF < 5%) and common indel variants (MAF > 5%), whereas YRI showed enrichment of low frequency indels relative to the other populations (Figure S1 in Additional file 1). As expected, the MAF distribution for indels was consistent with SNP site frequency spectra that revealed historical out-of-Africa bottlenecks for CEU and CHB+JPT [27].

#### Size distributions of indels revealed possible ascertainment bias in 1000G

1000G indels had size distributions similar to those previously found by Mills *et al*. [1] and Bhangale *et al*. [6]. Non-LOF indels ≤ 6 bp (small indels) represented approximately 90% of total indels (Figure S2a in Additional file 1). However, small indels were not distributed equally between low frequency and common (MAF > 5%) MAF bins. Small indels were more prevalent than indels > 6 bp (large indels) amongst low frequency indels by 20.6% (21.3% versus 16.9%, *P *< 2.2 × 10^-16^), whereas large indels were 5% more prevalent amongst common variants (82.9% versus 78.7%, *P *< 2.2 × 10^-16^) (Table S1 in Additional file 1). This finding, we reasoned, could be predominantly ascribed to ascertainment bias (due to short read NGS and alignment algorithms) that preferentially detected with confidence short length low frequency indels versus long length low frequency indel variants. However, we could not rule out biological reasons.

We initially suspected that the cause for the distribution skew might be due to large indels being more common amongst segmental duplication regions. However, after annotating for indel location using ANNOVAR annotation software [28], our results showed, on the contrary, that large common indels were less concentrated amongst segmental duplications than small ones (2.39% versus 2.67%, *P *= 6.328 × 10^-5^; Table S1 in Additional file 1). As such, we conclude that this difference is primarily due to ascertainment bias, which resulted from challenges in mapping repetitive regions.

### Global linkage disequilibrium patterns of INDELs

#### 1000G SNP LD patterns are comparable to previously published results

In our control analysis, we first evaluated SNP to SNP (SNP-SNP) LD using pairwise correlation coefficient r^2 ^with 1000G genotypes at 1000G, OMNI and HapMap SNP sites [29] (Materials and methods). HapMap SNP sites were discovered in the HapMap Phase 3 project while OMNI SNP sites were selected by Illumina from the 1000G genotypes due to their ability to tag common and rare variants while minimizing the amount of SNPs that needed to be genotyped [30]. We found that we were able recapitulate previously published SNP LD HapMap results (Table S3 in Additional file 1).

#### Indel-SNP LD was similar to SNP-SNP LD

Although SNPs and indels arise by different mechanisms in DNA replication or repair, previous studies using only common indels and SNP variants found that these polymorphisms had similar LD profiles, thus implying a shared evolutionary history [27,31]. Using the larger dataset provided by 1000G that includes both common and low frequency variants, we compared the LD properties between pairwise indel-SNP and SNP-SNP. Our results showed that indel-SNP and SNP-SNP LD profiles are similar to each other when calculated by average r^2 ^(Figure S3a in Additional file 1). This similarity was maintained when using the SNP sites from the 1000G, OMNI or HapMap panels. As discussed earlier, however, the similarity in indel-SNP and SNP-SNP LD profiles may be due to ascertainment bias - that is, highly polymorphic indel sites are not included in the dataset.

Not surprisingly, LD profiles differed based on the different SNP panels. The indel-SNP and SNP-SNP LD when using OMNI SNP sites had the lowest LD as the OMNI chip was specifically designed to minimize SNP redundancy. HapMap sites provided the highest indel-SNP LD because the panel had a relative dearth of low frequency SNP sites that would otherwise lower average indel-SNP LD. In contrast, the reduction in LD due to low frequency SNP sites was seen clearly in the 1000G panel when compared to HapMap (Figure S3a in Additional file 1). Lastly, indel-SNP LD echoed population-based SNP-SNP LD patterns; we found that YRI had lower LD than the other continental populations (Figure S3b in Additional file 1) due to the greater number of low frequency variants in that population.

#### Indels are well tagged by nearby SNPs

**Figure 1:**
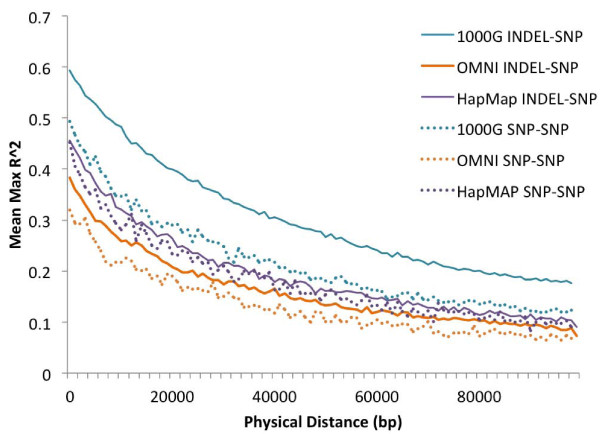
**Linkage disequilibrium pattern comparisons showed that INDEL-SNP LD is greater than SNP-SNP LD for mean maximum r^2^**. Mean max r^2 ^for the CEU population (CHB+JPT and YRI not shown) using common SNP and indel sites from 1000G, OMNI and HapMap, averaged over 1 kb windows. Although indel-SNP LD was higher than SNP-SNP for all SNP panels, this is likely due to ascertainment bias. OMNI LD was the lowest due to its design, and HapMap3 had the highest LD due to the relative reduction in low frequency variants.

**Figure 2:**
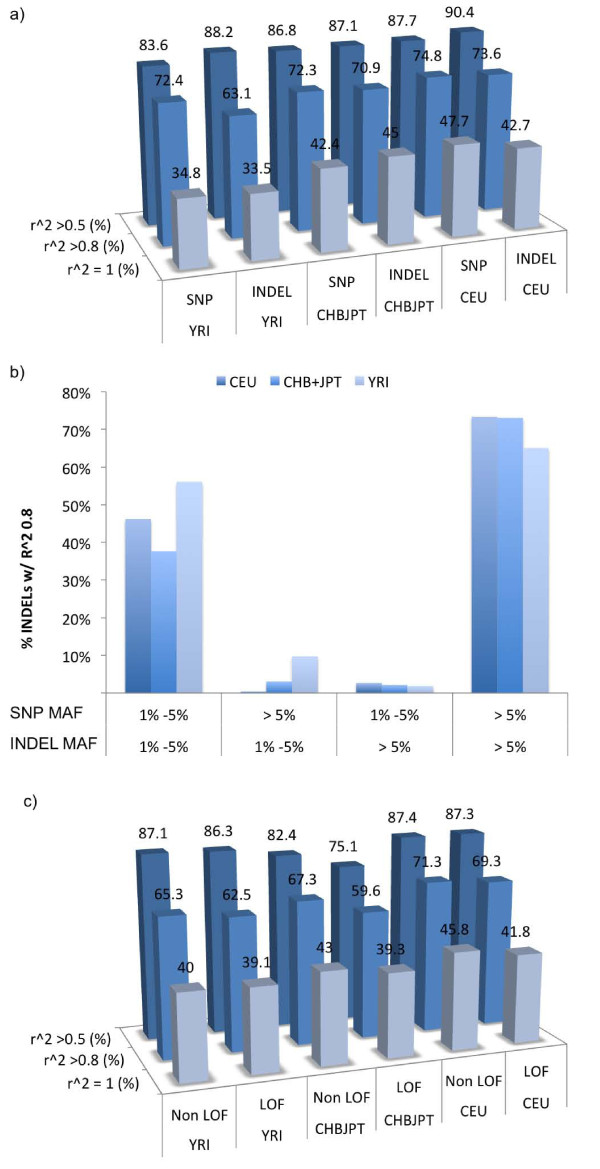
**Indels and SNPs show similar tag SNP patterns**. **(a) **We evaluated the percentage of common indels or SNPs with at least one pairwise r^2 ^greater than 0.5 (medium LD), 0.8 (high LD) or equal to 1 (perfect LD) using 1000G SNPs. Our results suggest that indels and SNPs are similarly tagged by nearby SNPs. **(b) **The percentage of low frequency and common indels that are in high LD with low frequency or common 1000G SNPs for CEU, CHBJPT and YRI populations. Low frequency and common indels are best tagged by frequency matching SNPs. **(c) **We evaluated tagging of LOF and non-LOF indels. The percentage of LOF that are in medium, high and perfect LD is slightly lower than non-LOF variants for all populations.

To explore indel tagging by nearby SNPs, we employed 'mean max r^2^', a measurement that averages the maximum r^2 ^values at given distances for putative alleles. The HapMap consortium used this measurement to describe SNP tagging [29]. Surprisingly, mean max r^2 ^for indel-SNP was significantly higher than SNP-SNP (Figure 1) for all SNP panels at all distances (*P *< 0.05, Mann-Whitney). Upon closer examination, we found that each indel has a larger number of SNPs (27.5) in high LD (r^2 ^> 0.80) when compared to the number of SNPs (16.15) in high LD with nearby SNPs. As there is a higher proportion of common variants amongst indels relative to SNPs (76.5% versus 65.2% averaged across all populations; Table S1 and Table S2 in Additional file 1), these data suggest that the inability to detect highly polymorphic and/or low frequency indels, which would not be tagged by nearby SNPs, may inflate the mean max r^2^. This is further corroborated by evidence that the percentage of indels having at least a single SNP in high LD was not different than the percentage of SNPs in high LD with at least one other SNP (Figure 2a). This pattern was also seen in CHB+JPT and YRI populations (data not shown). These data suggested that tagging of indels by SNPs is likely similar to tagging of SNPs; further, differences in mean max r^2 ^between indels and SNPs may be due to ascertainment bias.

Mean max r^2 ^values also reflected population differences in indel tagging. YRI showed lower indel-SNP LD when compared to CEU and CHB+JPT (Figure S3c in Additional file 1); common indels in YRI (63.12%) are not as well tagged as CEU (73.58%) and CHB+JPT (70.92%) due to greater diversity in the YRI population (Table S4 in Additional file 1). However, because of the relative increase in low frequency SNPs in the YRI population, low frequency YRI indels (60.99%) were better tagged than both CEU (47.30%) and CHB+JPT (39.53%) by 1000G SNPs (Table S4 in Additional file 1).

Lastly, we noted that OMNI and HapMap panels were also able to tag low frequency and common indels. In CEU and CHB+JPT, approximately 67% and approximately 66% of common indels were in high LD with at least one SNP using OMNI and HapMap panels, respectively. This is especially encouraging as we may be able to retrospectively impute associations with indels using existing GWAS datasets. However, these panels tag low frequency indels with lower efficiency: only 34.24% and 27.09% of low frequency indels in OMNI and HapMap panels, respectively, were in high LD with nearby SNPs (Table S4 in Additional file 1).

#### Strong LD between frequency-matched indels and SNPs

As common indels and SNP variants were previously found to have similar LD profiles [26,31], we predicted that low frequency indels and SNPs would also be similar. We found indel-SNP LD was primarily with frequency matched SNPs - that is, low frequency indels were found to be in LD with low frequency SNPs but not common SNPs (Figure 2b). For low frequency indels, YRI and CEU also show a greater percentage of indels in high LD (56.8% and 46.2%, respectively) with nearby SNPs relative to CHB+JPT (37.6%) (Figure 2b). This is due to the higher amounts of low frequency variants in YRI and CEU (Table S2 in Additional file 1). Similarly, common indels were in high LD with common SNPs but not with low frequency SNPs (Figure 2b). Overall, common indels are tagged at similar levels across populations by common 1000G SNPs (*P *> 0.05, all populations).

Further, as low-frequency indels likely represent recent mutations, the average length of extended haplotype should be longer for low frequency indels than for common indels [32]. As expected, median haplotype length for low frequency indels in high LD (31.6 kb) was longer than the median haplotype length for common indels in high LD (25.2 kb, *P *< 2.2 × 10^-16^) (Table S5 in Additional file 1). Meanwhile, there was no difference in haplotype lengths between common indels in high LD and common SNPs in high LD (*P *= 0.69) and between low frequency indels in high LD and low frequency SNPs in high LD (*P *= 0.19).

### Loss-of-function indels undergo purifying selection

LOF variants in the 1000G Pilot are defined as frame-shift, nonsense and splice site indels that may disrupt protein-coding regions [2]. Their deleterious nature causes these variants to undergo purifying selection; as such, they have lower MAF and shorter (and possibly less deleterious) lengths. In 1000G data, we observed evidence of this phenomenon in the MAF distribution and indel size distributions. Relative to non-LOF indels, LOF indels are enriched with low frequency variants; LOF indels within the MAF < 5% bin was approximately 50% greater than non-LOF indels (36.15% versus 23.51%, *P *< 2.2 × 10^-16^; Figure S1 in Additional file 1). Further LOF variants show a greater proportion of insertions and deletions of length one versus non-LOF populations (Figure S2a, b in Additional file 1).

Applying ANNOVAR to all indels revealed that indels are not evenly distributed amongst exonic and intronic/intergenic regions. In particular, low frequency CEU indels were more prevalent in exons when compared to common indels (0.24% versus 0.20%, respectively, *P *= 0.002; Figure S5a in Additional file 1). However, between low frequency and common non-LOF indels there was no difference in exonic prevalence (*P *= 0.36). This result suggests that deleterious variants were under purifying selection, thus decreasing the proportion of common variants in exons. This pattern was replicated in CHB+JPT and YRI populations (data not shown).

Our results also showed that 95% of LOF variants are small indels compared to 90% in non-LOF variants (*P *= 0.0017; Figure S2b in Additional file 1). Annotation by ANNOVAR revealed that a larger proportion of common large indels were located in exonic regions when compared to small indels (Figure S5b in Additional file 1; 0.28% versus 0.18%, *P *= 2.15 × 10^-8^). To investigate the cause, we partitioned exonic indels by functional impact (that is, by binning into LOF and non-LOF indels). Our analysis showed that LOF variants are more prevalent amongst exonic indels ≤ 6 bp (61.92%) when compared to exonic indels > 6 bp (48.81%, *P *= 0.00052). These findings provided further evidence of purifying selection; selection acts against larger (and potentially more deleterious) indels, thus decreasing the proportion of long LOF variants and increasing the proportion of benign indels in exonic regions. We found similar trends in both CHB+JPT and YRI populations (data not shown).

The relative increase in low frequency variants alters the LD profile of LOF variants in ways previously discussed. LOF variants had marginally lower LD for a given fixed distance between a putative indel and SNPs when compared to non-LOF variants because low frequency LOF variants had longer haplotypes (Figure S6a in Additional file 1). For distances less than 14 kbp, non-LOF variants had higher mean max r^2 ^than LOF variants (*P *< 0.05 for all comparisons). Low frequency variants also decrease the percentage of indels in high LD (r^2 ^> 0.80) with 1000G SNPs; there was a small but significant reduction of 1 to 4% in each population (Figure S6b in Additional file 1; *P *< 0.05 in all populations). LOF variants had slightly longer haplotype backgrounds than non-LOF variants due to the relative increase in low frequency indels amongst these alleles. Median haplotype length for LOF was approximately 2 kbp longer than non-LOF indels for all populations (*P *< 10^-10 ^for all populations; Table S6 in Additional file 1).

### Neighboring SNPs can accurately impute low frequency and common indels

Although the price of whole genome sequencing continues to drop, imputation of genotypes from SNP microarrays will continue to remain important for the foreseeable future. Statistical imputation of indels from SNPs is important and necessary to increase power for detecting variants in disease studies and central to the design of future SNP genotyping studies. With the large panel of SNP and indel genotypes provided by the 1000G, we assessed the effect of various population genetic properties, such as ethnicity, MAF bins of the indel, indel size, and LOF status, on the imputation power of indels. We also studied the importance of SNP density in imputation power and accuracy by comparing imputation results using three SNPs panels: 1000G, OMNI and HapMap3.

We evaluated power to impute homozygous reference (ref/ref), heterzygous (ref/alt) and homozygous alternative (alt/alt) alleles using two different imputation engines, SNPTools developed at the Baylor College of Medicine [32] and IMPUTE2 [3]. As SNPTools' imputation provided a > 15% improvement in imputation concordance when compared to IMPUTE2 (Table S7a, b in Additional file 1), we present only results generated with SNPTools in the main text. IMPUTE2 results and a detailed comparison are summarized in Additional file 1.

**Figure 3:**
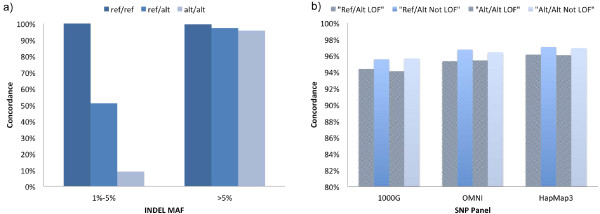
**Indels are imputed by nearby SNPs**. **(a) **Using SNPTools we imputed low frequency and common indels. For low frequency variants we achieved greater than 50% and 10% concordance for alt/alt and ref/alt imputation, respectively, and near 98% concordance for all common indels. **(b) **Imputations of ref/alt and alt/alt alleles for LOF variants were marginally harder to impute than non-LOF variants irrespective of SNP panel. Concordance rates were averaged over all populations.

As discussed in Figure 2b, common indels were well tagged by nearby common SNPs. As such we were not surprised that the concordance rate for imputing common indels was high. With SNPTools, imputation power was greater than 96% for ref/ref, ref/alt and alt/alt imputations in all populations (Figure 3a). This high level of accuracy was maintained when using SNPs from 1000G, OMNI or HapMap panels (Table S8a in Additional file 1).

#### 1000 Genomes SNPs imputed low frequency heterozygous indels with greater than 50% concordance but could not impute accurately homozygous alternative alleles

Perhaps of greater interest due to the impact on disease phenotypes by low frequency variants [33-36], we explored imputation of low frequency alleles from 1000G SNPs. We found that SNPTools ably imputed heterozygous low frequency alleles. Using population averages, ref/alt alleles were imputed with a 50.0% concordance rate while alt/alt alleles were imputed with an approximately 11.9% concordance rate (Figure 3a) from 1000G SNPs. In addition, YRI demonstrated superior imputation power for ref/alt (50.3%) when compared to CHB+JPT (44.5%) but not when compared to CEU (52.2%) (Table S8a in Additional file 1). This is likely due to the greater percentage of low frequency indels in the YRI population that are in high LD with nearby SNPs (Table S5a in Additional file 1).

#### Imputation of LOF variants was similar to non-LOF variants

As LOF indels represent possible deleterious variants, confidence in imputing LOF indels is of interest to GWAS studies. As there was a higher proportion of low frequency amongst LOF variants relative to non-LOF variants (Figures S1 in Additional file 1), overall imputation concordance for all (common + low frequency) variants suffers. For ref/alt and alt/alt variants, imputation concordance for all (common + low frequency) non-LOF variants was greater than 95%. Ref/alt and alt/alt concordance for LOF variants was approximately 1 to 2% lower in all populations when compared to non-LOF variants (*P *< < 0.05 for all comparisons) (Figure 3b). The small reduction in imputation power between non-LOF and LOF variants was due to differences in LD contingent on their respective MAF distributions (Tables S7b and S8b in Additional file 1).

### Increased r^2 ^improved imputation performance in SNPTools' indel imputation

Imputation of genotypes in unrelated individuals utilizes the shared LD structure of the population. To explore the impact of LD on imputation power, we created bins of SNPs with r^2 ^values from zero to one in 0.05 increments. We then used these bins to iteratively impute indel variants.

**Figure 4:**
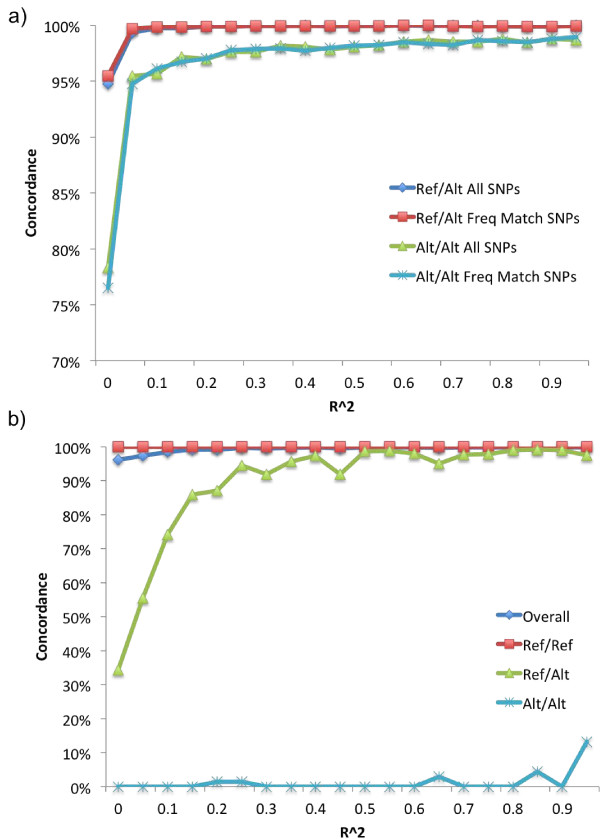
**Imputation performance depends on r^2 ^of nearby SNPs**. **(a) **For common indels, frequency matched SNP imputation performs similar to imputation using all SNPs. **(b) **For low frequency indels, imputation performance improves rapidly for heterozygous (ref/alt) imputation with > 90% concordance once r^2 ^between indels and SNPs reaches 0.25. However, imputation of alternative homozygous alleles (alt/alt) remains below 15% even when imputed with SNPs in perfect LD.

In Figure 2b above, we reported that indel LD was highest between frequency-matched indels and SNPs. Correspondingly, we evaluated if we could obtain equivalent imputation performance and reduce computational requirements by using only frequency-matching SNPs. As expected, our results showed that, for common indels, we could obtain similar power to impute ref/alt and alt/alt when using only frequency matching SNPs (Figure 4a).

For low frequency indels we found that higher LD makes a large impact on imputation performance. Overall imputation performance closely tracked ref/ref concordance due to the overall preponderance of ref/ref imputations (approximately 93% of all comparisons) relative to ref/alt imputations (approximately 6% of all comparisons) and alt/alt imputations (approximately 1% of all comparisons). For imputation of heterozygous alleles there was a rapid increase in concordance from approximately 35% when using SNPs with r^2 ^values between 0 and 0.05 to greater than 90% concordance for r^2 ^greater than 0.25 (Figure 4b). Unfortunately, we still found that there was little power to impute homozygous alternative alleles. Results were replicated in YRI and CHB+JPT populations (data not shown).

## Discussion and conclusion

The 1000G Consortium [2], Mills *et al*. [1,7,33], Bhangale *et al*. [6] and other studies previously reported that short indels are not only abundant in the genome but also likely to impact human phenotypic diversity. In the 1000G dataset, the CEU, CHB+JPT and YRI populations had 1,316, 1,694, and 1,691 indels, respectively, that resided in exonic regions, of which 578, 540, and 804 of these variants, respectively, were predicted to be LOF variants (Figure S3 in Additional file 1).

Given the impact of LOF variants on genetic function, our study revealed that LOF variants likely undergo purifying selection (Figures S1, S3a, S3b, and S6, and Table S6 in Additional file 1) [1]. Of particular interest to human health, LOF variants were nearly as well tagged by nearby SNPs as non-LOF variants (Figure S6b in Additional file 1). Further, while previous studies by Frazer *et al*. [34], Eichler *et al*. [35], and McCarroll *et al*. [4,36] demonstrated that common indels are in high LD with nearby SNPs, our results revealed that both low frequency and common indels can be reliably tagged by nearby SNPs (Figure 2a). OMNI and HapMap panels ably tag > 70% of common indels, while OMNI tags > 50% of low-frequency indels (Table S4 in Additional file 1) in all populations.

In addition to evaluating LD, we provided in our study descriptive statistics of imputation performance. By retroactively imputing indels from high scoring loci in previous GWAS, it may be possible to identify previously unknown causative variants. Our internal imputation engine SNPTools can impute greater than 95% of common indels in all populations. However, we found that imputation of low frequency was more difficult as fewer indels are in high LD with nearby SNPs (Table S4 in Additional file 1). It has been assumed that low frequency variants, which provide a large proportion of inherited susceptibility to disease [37], cannot be easily imputed due to their low MAF. We find this previous statement to be partially true; while we could impute heterozygous alleles with 50% concordance, we had very little power to impute homozygous alleles. Our results show that improvements in imputing low-frequency indels in GWAS results can be gained by including more low frequency 'tag-SNPs' in post 1000G microarray panels. However, as LD amongst low-frequency indels and low-frequency SNPs was not as high as that between common indels and common SNPs, such advantages will be accretive. As we can use current imputation engines to impute indels from previously published or future GWAS, we provide to the community a list of indels tagged with nearby SNPs with high LD (r^2 ^> 0.8) [25].

In this study we evaluated the LD patterns and imputation results for indels called from low coverage NGS technology. Unfortunately we could not evaluate indel calls from high coverage exon capture due to the relative difficulty in calling indels from exonic data and the relative lack of data in the 1000G Exon Pilot 3 [38,39]. However, we do expect that these data will be important in validating future indel calls. To date, calling short indels from short read NGS data remains technically challenging; however, this method may provide greater fidelity than trace mapping for low frequency and small indels. While future work comparing and validating these various approaches is necessary, these complementary technologies nonetheless help us achieve the ultimate goal of generating a comprehensive understanding of indels and their impact on human variation and disease.

## Materials and methods

### Data preparation

We used short indels (< 50 bp) and SNP genotype data from 1000G Pilot 1 (sequenced to approximately 3.6X coverage) from the October 2010 Pilot paper data set releases available in VCFv4 format for CEU, YRI and CHB+JPT populations [40]. There were 60 CEU, 59 YRI and 60 CHB+JPT samples. Indels were called using Dindel [39], which accounts for difficulties in mapping, alignment, and size using a bayesian framework. In the 1000G Pilot Paper and Supplement, the false discovery rate for novel variants was reported to be 1.7% for low-coverage indels [2]. This was achieved by using a stringent filtering scheme that took into account mapping quality, number of reads and QCALL imputation estimates. Novel indels (not found in dbSNP 129) were chosen from chromosome 20 and genotyped using Sequenom; 79, 59, and 152 indels were genotyped from the CEU, CHB+JPT, and YRI populations, and the true positive rate) was found to be 98.7%, 94.9% and 99.3%, respectively (Table S3 and Supplemental information in Additional file 1) [2].

The SNPs and indels were merged using a custom C++ script. During the merging process, if an indel or SNP shared the same position, the indel was retained and the SNP was discarded. In addition, we only evaluated bi-allelic indels and SNPs. Multi-allelic polymorphisms (SNPs with multiple alternative alleles) represented approximately 0.01% of total SNPs, and were not included in our current study.

A list of predicted LOF indel sites was also released in October 2010 with the pilot paper [40]. These LOF variants represent predicted protein disrupting variants [2,38]. Data manipulation was done with custom PERL scripts. We removed all rare indels (MAF < 1%), as all incidences were singletons. Data sparsity prevented us from drawing any conclusions about their characteristics.

To generate OMNI and HapMap genotype panels, the 1000G genotypes were filtered using OMNI and HapMap Phase 3 sites, which can be obtained from Illumina [30] and the International HapMap Project website [29,41], respectively.

### Linkage disequilibrium

Indels were sampled using the sampler program. Correlation coefficient (r^2^) was calculated using PLINK (version 1.7 x86_64) [42]. Using PLINK, pairwise correlation coefficients were calculated for each indel and each SNP for a ± 100 kb region of the indel. Evaluations of SNP to SNP pairwise correlation coefficients were completed with > 1,500 randomly sampled SNPs from OMNI, 1000G and HapMap3 panels. All charts comparing r^2 ^to physical nucleotide distance (base pairs) were completed using custom scripts on the statistical software package R (version 2.12.0) and graphed in EXCEL (MS Office for Windows 2007). Mean max r^2 ^was calculated by averaging the maximum r^2 ^values for all indels within 1 kb windows. Average r^2 ^was calculated by averaging all r^2 ^values within 100 bp windows. Statistical tests are indicated in the manuscript.

### Imputation

Two LD-based imputation engines were chosen to provide more robust imputation of indels. Imputation was completed with both IMPUTE2 (version 2) [3] and the imputation engine from SNPTools (manuscript under review). Indels were first sampled using the aforementioned sampler program, and the indel was imputed using all SNPs within a ± 100 kb region. Concordance was calculated by summing over k indels and then summing over imputed n_1 _individuals the count of correctly imputed genotypes. The genotypes for both the indels and SNPs provided by the 1000G Pilot project were assumed to be correct. In the SNPTools imputation engine, these genotypes were given a genotype likelihood of 0.995.

Concordance was calculated separately for imputed genotypes for homozygous reference (ref/ref), heterozygous (ref/alt) and homozygous alternative (alt/alt). Concordance rates for populations and MAF were graphed using EXCEL.

To evaluate the effects of LD on imputation, the pairwise r^2 ^was first calculated for all SNPs within ± 100 kbp of 1,500 indels. These SNPs were grouped by pairwise r^2 ^values, in 0.05 increments, that is, 0 to 0.05, 0.05 to 0.10 ... 0.95 to 1. Each grouping of r^2 ^was then used to impute the indel genotype. Concordance rates were then calculated.

### Statistical analyses

Paired comparison of count information/proportions was completed using the two-proportion test. Population comparisons for indel tagging and haplotype length were evaluated using the Mann-Whitney test.

## Abbreviations

1000G: 1000 Genomes Project; bp: base pair; GWAS: genome-wide association studies; indel: insertion-deletion; kbp: kilo-base pairs; LD: linkage disequilibrium; LOF: loss-of-function; MAF: minor allele frequency; NGS: next generation sequencing; SNP: single nucleotide polymorphism.

## Competing interests

The authors declare that they have no competing interests.

## Authors' contributions

JTL and FY conceived the project. JTL completed the analysis; YW assisted with analysis. JTL, YW, FY, and RAG wrote, reviewed and approved the manuscript.

## Supplementary Material

Additional file 1 **Supplemental material**. Supplemental file includes nine tables and seven figures. Click here for file
