# Supplementary material for: Characterizing linkage disequilibrium and evaluating imputation power of human genomic insertion-deletion polymorphisms
Source: Genome Biol. 2012 Feb 29;13(2):R15. doi: 10.1186/gb-2012-13-2-r15 (PMC3334570; doi:10.1186/gb-2012-13-2-r15)
Supplement: Additional file 1 — Supplemental material. Supplemental file includes nine tables and seven figures. [file gb-2012-13-2-r15-S1.DOC]

Supplementary Information

Table of Contents:

1. Analysis of 1000G INDELs
   1. Figure S1: Minor allele frequency (MAF) distributions for each population.
   2. Table S1: Minor allele frequency distribution by population
   3. Figure S2a: INDEL Size Distribution for non-LOF variants
   4. Figure S2b: INDEL Size Distribution for LOF variants
   5. Table S2: Percentage of CEU INDELs ≤6 bp and > 6p by Minor Allele Frequency
2. SNP-SNP LD Patterns
   1. Table S3: Counts of SNPs by MAF for 1000 Genomes Low Coverage Pilot
   2. Table S4: Percentage of SNPS with at least one pairwise r^2^ value with another SNP by population and panel
3. INDEL-SNP LD Patterns
   1. Figure S3a: Average r^2^ for SNP-SNP and INDEL-SNP genotypes for CEU
   2. Figure S3b: Average r^2^ for SNP-SNP and INDEL-SNP genotypes for all populations evaluated with 1000G sites
   3. Table S5a: Percentage of INDELs with at least one pairwise r^2^ value with a nearby SNP by population and panel.
   4. Table S5b: Physical distance between rare, low frequency, and common INDEL-SNP with high LD (r^2^>0.80).
   5. Figure S4a: Mean max r^2^ for common short (≤6 bp) and long (7-50 bp) CEU INDELs with 1000G SNPs
   6. Figure S4b: Mean max r^2^ for common short (≤6 bp) and long (7-50 bp) CEU insertions and deletions
4. LOF INDELs undergo purifying selection
   1. Figure S5a: INDEL annotation with ANNOVAR
   2. Figure S5b: Comparison of INDELs ≤ 6 bp and INDELs >6 bp.
   3. Figure S6a: Plot of mean max INDEL to SNP pairwise r^2^ using 1 kbp windows for LOF and non LOF CEU with 1000G SNPs
   4. Figure S6b: Percentage of LOF and non-LOF INDELs with at least one pairwise r^2^ value ≥ 0.5 (medium LD), ≥ 0.8 (high LD) or equal to 1 (perfect LD) when using 1000G SNPs
   5. Table S6: Physical distance between LOF and non LOF INDELs to nearby SNPs that have high LD (r^2^ >0.8).
5. Imputation performance with SNPTools is more accurate than Impute2
   1. Table S7a: Comparison of Imputation performance of IMPUTE2 and SNPTOOLS for CEU
   2. Table S7b: Comparison of Imputation performance of IMPUTE2 and SNPTOOLS for CEU LOF INDELs
   3. Table S8a: SNPTool imputation concordance for low frequency and common INDELs imputed with 1000G, OMNI and HapMap panels for each population
   4. Table S8b: SNPTool imputation concordance for LOF and non-LOF INDELs imputed with 1000G, OMNI and HapMap panels for each population
   5. Table S8c: Comparison of imputation concordance for low frequency INDELs and low frequency SNPs using SNPTOOLS
   6. Table S9a: IMPUTE2 imputation concordance for low frequency and common INDELs imputed with 1000G for each population
   7. Table S9b: IMPUTE2 imputation concordance for LOF and non-LOF INDELs imputed with 1000G, OMNI and HapMap panels for each population
6. INDELs ≤ 6 bp are imputed with marginally higher accuracy than INDELs >6 bp
   1. Figure S7: Imputation performance for low frequency and common INDELs ≤ 6 bp and >6 bp

# 1) Analysis of 1000G INDELs

The 1000 Genomes Project ([http://www.1000genomes.org](http://www.google.com/url?q=http%3A%2F%2Fwww.1000genomes.org&sa=D&sntz=1&usg=AFQjCNHUCxx0uzrYAJySeGo7aPcUc_sDiQ)) was composed of three pilot projects representing alternative coverage strategies. In this study, we utilized data from Pilot 1: low-pass sequencing of 179 individuals at ~2-4 X read-depth. This pilot was aimed to enable discovery of a wide variety of genetic polymorphisms. We focused primarily on INDEL variant calls.

In this section, we explored the general characteristics of these INDELs. Key results were discussed in the main text.


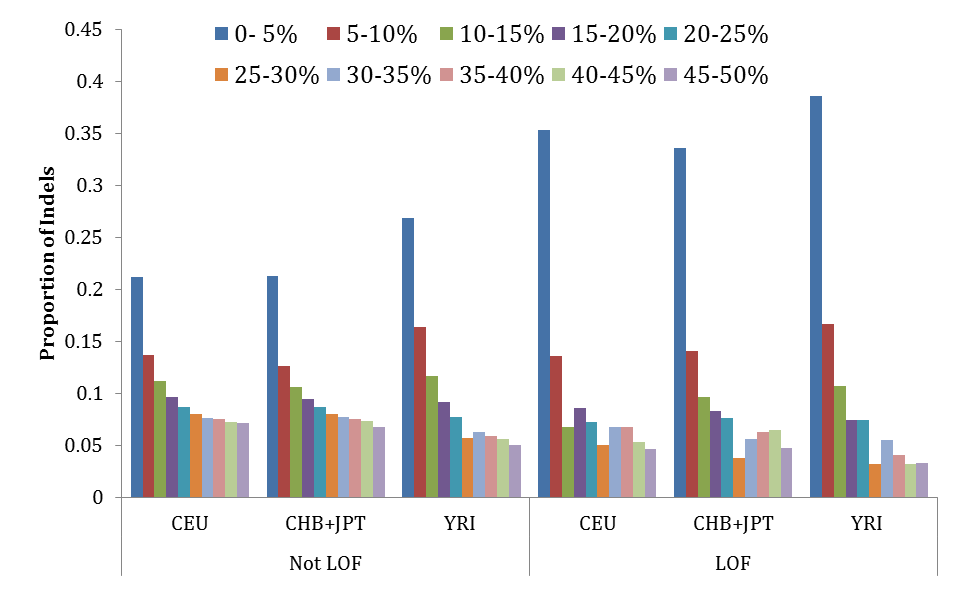


**Figure S1: Minor allele frequency (MAF) distributions for each population.** There are two main observations: (1) there was an enrichment of low frequency INDELs (MAF < 5%) in the YRI population and (2) there was a greater proportion of low frequency INDELs amongst LOF INDELs; suggesting purifying selection of LOF INDELs.

|  | CEU | CHB+JPT | YRI |
| --- | --- | --- | --- |
| Samples | 60 | 60 | 59 |
| # INDELs < 1% MAF | 62,231 (8.54%) | 62,744 (9.41%) | 74,838 (7.94%) |
| # INDELs 1-5% MAF | 92,436 (12.69%) | 79,520 (11.92%) | 177,945 (18.89%) |
| # INDELs >5% MAF | 573,864 (78.77%) | 524,822 (78.67%) | 689,419 (73.17%) |

**Table S1: Minor allele frequency distribution by population**


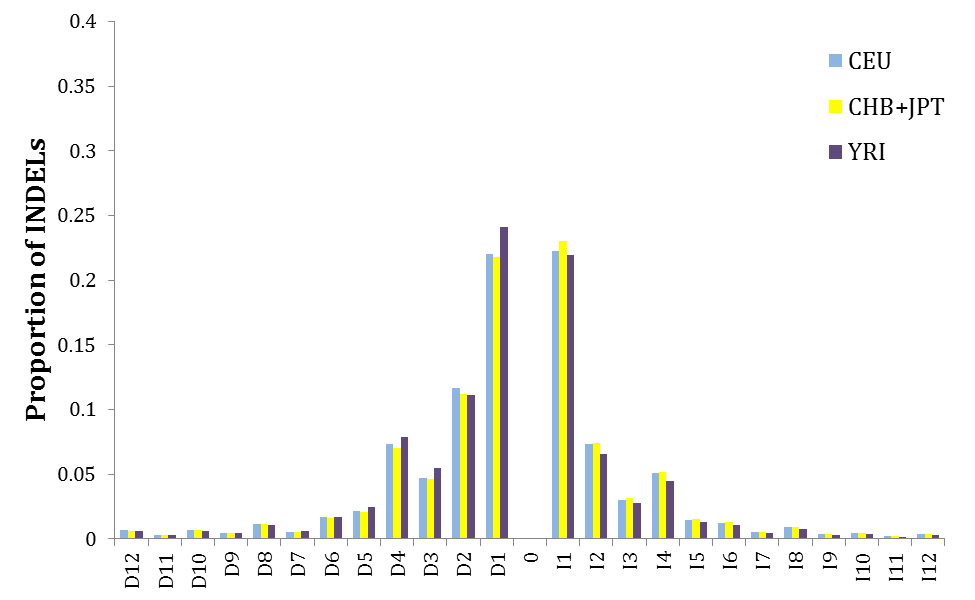


**Figure S2a: INDEL Size Distribution for non-LOF variants:** The plot shows INDEL size distribution for all populations. Approximately 90% of INDELs were less than 6 bp in length. INDELs less than 12 bp represent 95% of all INDELs. The max INDEL length in the dataset was 48 bp.


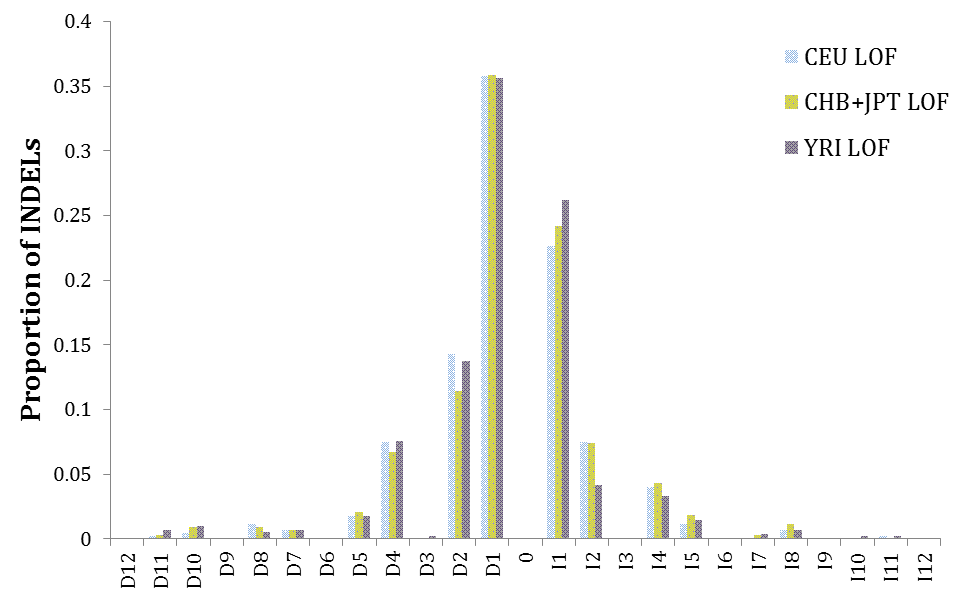


**Figure S2b: INDEL Size Distribution for LOF variants:** The plot shows INDEL size distribution for all populations. Approximately 95% of INDELs were ≤ 6 bp in length. INDELs less than 12 bp represent over 98% of all INDELs. The max INDEL length in the dataset was 47 bp.

| **% of INDELs** | | **MAF** | |
| --- | --- | --- | --- |
|  |  | 0.01-0.05 | 0.05-0.50 |
| **INDEL SIZE** | 1-6 | *21.32%* | *78.54%* |
|  | 7-50 | *16.90%* | *82.92%* |
| **% of these INDELs in segmental duplication** | | **MAF** | |
|  |  | 0.01-0.05 | 0.05-0.50 |
| **INDEL SIZE** | 1-6 | *2.32%* | *2.78%* |
|  | 7-50 | *2.34%* | *2.41%* |

**Table S2: Percentage of CEU INDELs ≤6 bp and > 6p by Minor Allele Frequency:** The percentage of INDELs >6 bp is greater for INDELs with MAF > 5%. Corresponding INDELs ≤6 bp are more prevalent among INDELs with MAF between 1-5%. This could not be explained by the percentage of these INDELs in segmental duplication regions. Rather this result may be due to ascertainment bias in short read length next generation sequencing. Analysis of YRI and CHB+JPT were similar.

# 2) SNP-SNP LD Patterns

In this section, we evaluate the LD characteristics of SNPs found in the 1000G Low Coverage Pilot. Increased SNP density in 1000G provided greater coverage of rare and low frequency SNPs. The 1000 Genomes re-sequencing data has significantly overcome the frequency related ascertainment bias issue in the HapMap project, resulting in an enriched collection of SNPs in the lower end of the frequency spectrum, closely concordant with the neutrality expectation[2]. Specifically, the 1000 Genomes panel has roughly 200 and 20 fold more SNPs in rare and low frequency bins than HapMap (Table S2).

|  | **1000 G** | | | **OMNI** | | | **HapMap3** | | |
| --- | --- | --- | --- | --- | --- | --- | --- | --- | --- |
|  | CEU | CHBJPT | YRI | CEU | CHBJPT | YRI | CEU | CHBJPT | YRI |
| Total | 7,699,035 | 6,092374 | 1,052,6297 | 1,520,848 | 1,276,611 | 1,839,581 | 1,181,544 | 1,083,713 | 1,237,670 |
| 0-0.01 | 1,063,903 (13.8%) | 486,252 (8.0%) | 2,424,114 (23.0%) | 74,735 (4.9%) | 27,061 (2.1%) | 208,692 (11.3%) | 2,522 (0.2%) | 1,349 (0.1%) | 11,341 (0.9%) |
| 0.01-0.05 | 1,172,299 (15.2%) | 613,889 (10.1%) | 2,699,672 (25.6%) | 302,275 (19.9%) | 79,404 (6.2%) | 375,635 (20.4%) | 47,682 (4.0%) | 26,771 (2.5%) | 113,167 (9.1%) |
| 0.05-0.50 | 5,462,833 (71.0%) | 4,992,233 (81.9%) | 5,402,511 (51.3%) | 1,846,723 (75.2%) | 1,170,146 (91.7%) | 1,255,254 (68.2%) | 1,131,339 (95.8%) | 1,055,593 (97.4%) | 1,113,162 (89.9%) |

**Table S3: Counts of SNPs by MAF for 1000 Genomes Low Coverage Pilot.**  SNPs were downsampled using OMNI and HapMap3 sites for the CEU, YRI, and CHBJPT populations.

Using average, we found that HapMap LD was greater than both 1000G and OMNI (Figure S4a). As the OMNI SNP panel was designed to minimize the usage of non-redundant SNPs, it was not surprising that these SNPs had the lowest LD. The high proportion of low frequency alleles in YRI due to the population's higher genetic diversity reduced LD in that population relative to the CEU and CHB+JPT populations (Figure S4b).

Like previous HapMap studies [3,4], SNPs were largely well tagged by other SNPs. 75-80% of all 1000 G SNPs in the three populations were in moderate LD (r^2^ >0.50) with at least a single other SNP, while 40-48% of SNPs had at least 1 SNP that was in perfect LD (r^2^ =1). As expected, larger SNP panels are better able to tag other SNPs; the percentage of SNPs in high LD (r^2^ >0.80) using 1000G SNPs (67.32%) was reduced when down sampled for OMNI (43.00%) and HapMap (57.26%) in CEU. (Table S3).

| Population | SNP Panel | % SNPS with r^2^ >0.5 | % SNPS with r^2^ >0.8 | % SNPS with r^2^ = 1 |
| --- | --- | --- | --- | --- |
| CEU | 1000G | 80.08% | 67.32% | 48.91% |
| CEU | OMNI | 66.41% | 43.00% | 21.45% |
| CEU | HapMap | 75.19% | 57.26% | 33.67% |
| CHBJPT | 1000G | 77.09% | 63.12% | 40.49% |
| CHBJPT | OMNI | 71.29% | 47.65% | 22.15% |
| CHBJPT | HapMap | 76.90% | 59.37% | 34.14% |
| YRI | 1000G | 80.97% | 59.57% | 40.94% |
| YRI | OMNI | 53.77% | 23.31% | 8.89% |
| YRI | HapMap | 56.52% | 34.44% | 17.95% |

**Table S4: Percentage of SNPS with at least one pairwise r^2^ value with another SNP by population and panel.** The percentage of SNPs with at least one pairwise r^2^ value greater than 0.5 (medium LD), 0.8 (high LD) or equal to 1 (perfect LD). While HapMap and OMNI SNPs provide some tagging of INDELs, the 1000G panel provides the best coverage.

To verify our results, we used datasets from HapMap Phase 1,2 and 3 (<http://hapmap.ncbi.nlm.nih.gov/>) and were able to able to recapitulate their results using our scripts in R.

# 3) INDEL – SNP LD Patterns

We next explored LD of INDEL with nearby SNPs. We evaluated INDELs by 1) the ethnicities, (2) MAF bins of the INDEL, (3) INDEL size, (4) and LOF status on LD of INDELs.

These figures are primarily discussed in the main text in comparison to mean max r^2^. We note that INDEL-SNP and SNP-SNP LD were similar for all panels, but there were differences in LD when using different SNP panels (Figure S3a and S3c). We also reiterated populated based differences in LD. Again, we find that CEU and CHB+JPT had higher LD than YRI due the greater genetic diversity in the YRI population.


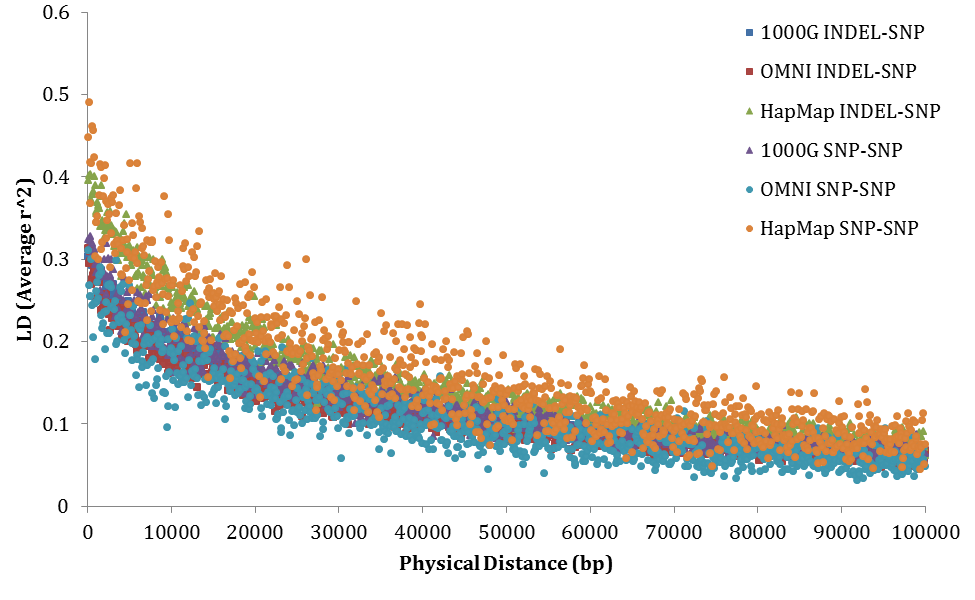


**Figure S3a: Average r^2^ for INDEL-SNP and SNP-SNP genotypes for CEU.** 1000G SNPs were downsampled for OMNI and HapMap sites. While there did not appear to be much difference between INDEL-SNP LD and SNP-SNP LD, there is significant difference between the different panels. OMNI had the lowest LD due to the design of the OMNI chip. 1000G LD is less than HapMap due to the increase in low frequency variants. (CHBJPT and YRI are similar to CEU). Average r^2^ was calculated using 100 bp window.


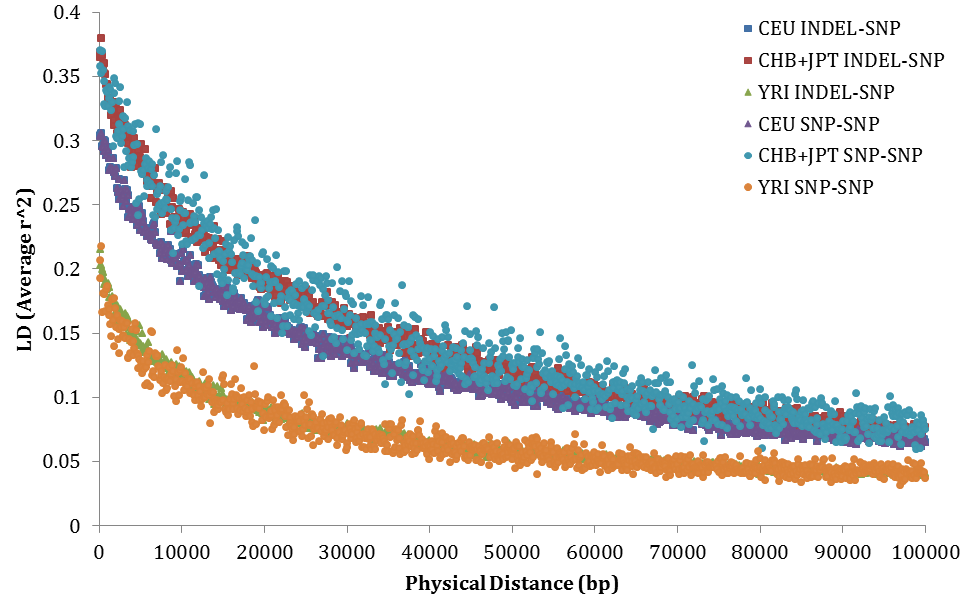


**Figure S3b: Average r^2^ for SNP-SNP and INDEL-SNP genotypes for all populations evaluated with 1000G sites.** INDEL-SNP had similar LD to SNP-SNP in all populations. The CHB+JPT population had the highest LD due to highest prevalence of common variants while YRI with the highest proportion of low frequency INDELs had the lowest LD. Average r^2^ was calculated using a 100 bp window.

Of interest is the percentage of INDELs with tagSNPs for a given SNP panel and population. In Table S4, we summarized the average of 2,000 putative INDELs for each combination of population and SNP panel.

| Population | MAF | PANEL | % INDELs with r^2^ >0.5 | % INDELs with r^2^>0.8 | % INDELs with r^2^ = 1 |
| --- | --- | --- | --- | --- | --- |
| CEU | 1% -5% | 1000G | 70.47% | 47.30% | 44.42% |
| CEU | 1% -5% | OMNI | 54.71% | 34.24% | 30.58% |
| CEU | 1% -5% | HAPMAP | 42.56% | 27.09% | 21.94% |
| CHB+JPT | 1% -5% | 1000G | 58.05% | 39.53% | 36.71% |
| CHB+JPT | 1% -5% | OMNI | 45.15% | 28.55% | 26.67% |
| CHB+JPT | 1% -5% | HAPMAP | 37.09% | 23.92% | 21.95% |
| YRI | 1% -5% | 1000G | 84.81% | 60.99% | 44.42% |
| YRI | 1% -5% | OMNI | 64.03% | 35.83% | 31.56% |
| YRI | 1% -5% | HAPMAP | 24.47% | 10.52% | 8.78% |
| CEU | > 5% | 1000G | 90.38% | 73.58% | 42.68% |
| CEU | > 5% | OMNI | 86.71% | 67.70% | 33.19% |
| CEU | > 5% | HAPMAP | 84.12% | 66.67% | 33.98% |
| CHB+JPT | > 5% | 1000G | 87.13% | 70.92% | 44.98% |
| CHB+JPT | > 5% | OMNI | 83.63% | 65.92% | 33.29% |
| CHB+JPT | > 5% | HAPMAP | 82.98% | 66.09% | 36.69% |
| YRI | > 5% | 1000G | 88.19% | 63.12% | 33.52% |
| YRI | > 5% | OMNI | 81.58% | 50.88% | 19.88% |
| YRI | > 5% | HAPMAP | 71.77% | 45.32% | 20.42% |
| CEU | ALL | 1000G | 86.27% | 69.48% | 43.30% |
| CEU | ALL | OMNI | 79.73% | 62.28% | 32.38% |
| CEU | ALL | HAPMAP | 77.69% | 59.15% | 32.00% |
| CHB+JPT | ALL | 1000G | 81.39% | 66.46% | 43.59% |
| CHB+JPT | ALL | OMNI | 77.36% | 61.40% | 35.01% |
| CHB+JPT | ALL | HAPMAP | 76.41% | 60.74% | 36.16% |
| YRI | ALL | 1000G | 87.55% | 64.11% | 39.54% |
| YRI | ALL | OMNI | 73.86% | 44.55% | 20.48% |
| YRI | ALL | HAPMAP | 61.74% | 38.44% | 18.86% |

**Table S5a: Percentage of INDELs with at least one pairwise r^2^ value with a nearby SNP by population and panel.**  Percentage of INDELs with at least one pairwise r^2^ value greater than 0.5 (medium LD), 0.8 (high LD) or equal to 1 (perfect LD) when using 1000G, OMNI or HapMap SNP panels. INDELs are also normalized by MAF.

The mechanisms for low frequency INDELs include recent mutations, selection pressure or under genetic drift. Recently formed INDELs are expected to have longer haplotypes because they have yet to undergo recombination. We found this to be true, low frequency INDELs (and SNPs) on show longer haplotypes than more common variants (Table S5).

| Population | INDEL MAF | Relationship | 1st Qu. ( bp) | Median ( bp) | 3rd Qu. ( bp) |
| --- | --- | --- | --- | --- | --- |
| CEU | 1% -5% | SNP-SNP | 20260 | 28590 | 44040 |
| CEU | > 5% | SNP-SNP | 17980 | 26570 | 40550 |
| CEU | 1% -5% | INDEL-SNP | 23780 | 31610 | 48620 |
| CEU | > 5% | INDEL-SNP | 15890 | 25180 | 37540 |

**Table S5b: Physical distance between rare, low frequency, and common INDEL-SNP with high LD (r^2^>0.80).** There was a significant difference in haplotype length between common and rare INDEL-SNP and SNP-SNP pairs respectively (Mann-Whitney test, p<0.05). A similar relationship was found with YRI and CHB+JPT.

*LD differences between common INDELs >6 bp and INDELs ≤ 6 bp is due to differences in LD for insertions vs. deletions*

We next explored whether INDELs >6 bp and INDELs ≤ 6 bp may have different LD characteristics. We choose 6 bp as the cutoff because that length encompasses over 90% of detected INDEL variants. Common INDELs ≤ 6 bp had higher LD than INDELs >6 bp (mean max r^2^ =0.61 vs. 0.57, p=0.026, Mann-Whitney) at a distance of 1 kbp but that difference narrowed as the distance between INDEL and SNP reached 100 kbp (Figure S4a). Low frequency INDELs did not have this pattern. Upon further examination, the difference in mean max r^2^ for INDELs ≤ 6 bp and INDELs >6 bp was found to be due to differences in LD for insertions (0.62 vs. 0.54, respectively, at 1 kbp, p =0.0031, Mann-Whitney) but not for deletions (p>0.05, Mann-Whitney) (Figure S4b).


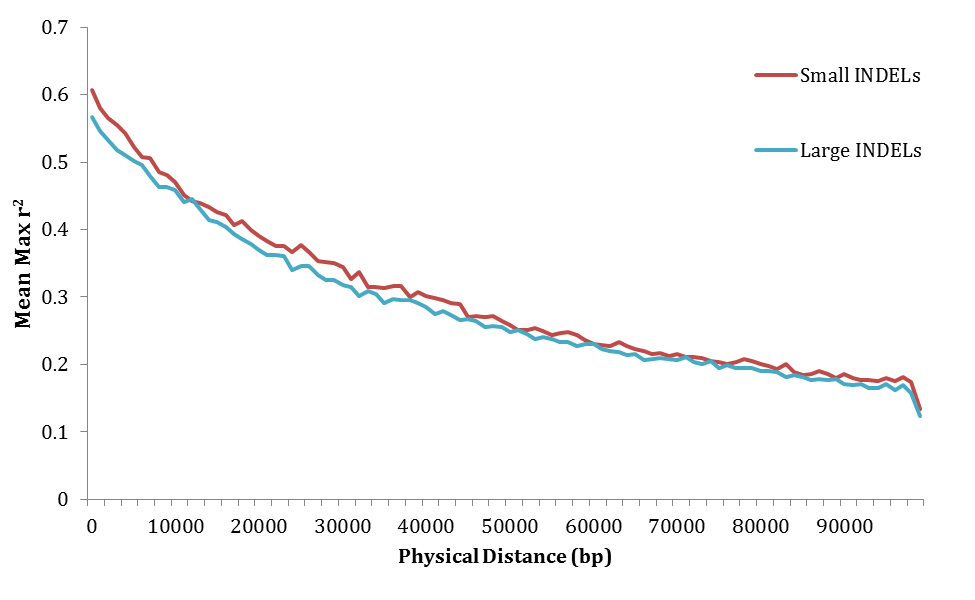


**Figure S4a: Mean max r^2^ for common short (≤6 bp) and long (7-50 bp) CEU INDELs with 1000G SNPs.** Short INDELs have higher LD, with an increase r^2^ of 0.04 at physical distances less than 5 kbp, and 0.01 at 100 kbp. CHBJPT and YRI show similar patterns (data not shown). Mean max r^2^ is calculated using 1 kbp windows.


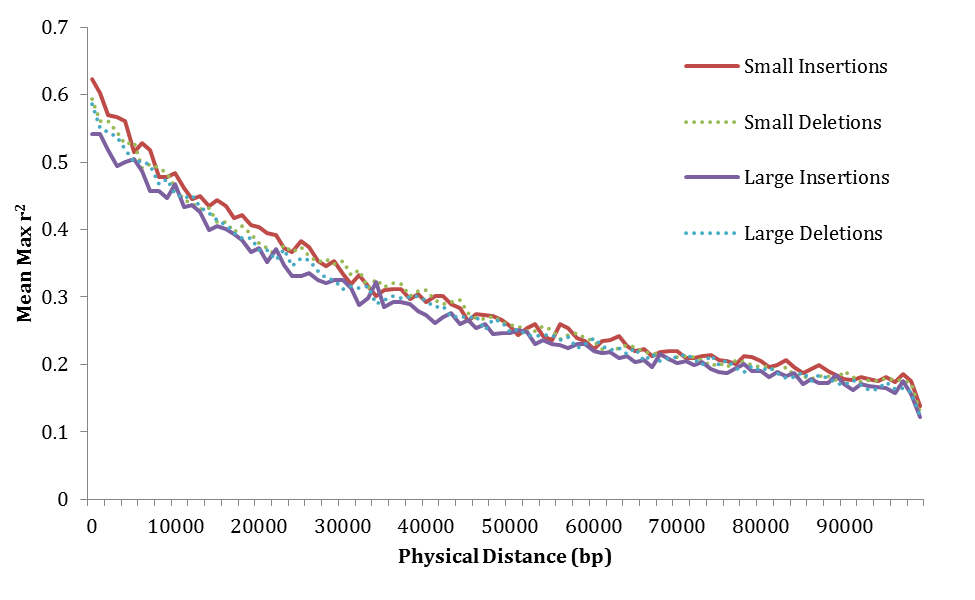
**Figure S4b: Mean max r^2^ for common short (≤6 bp) and long (7-50 bp) CEU insertions and deletions.** Small insertions have higher LD than small deletions, but there is no difference between large insertions or deletions. CHBJPT and YRI show similar patterns (data not shown).

# 4) LOF INDELs undergo purifying selection

We applied ANNOVAR [27] to all INDELs and found that ~0.18% (CEU and YRI) and ~0.25% (CHB+JPT) of INDELs lie in exonic regions, while the vast majority of INDELs lying in intergenic (~54%) and intronic (~32%) regions. Low frequency INDELs when compared to common INDELs were disproportionately enriched with exonic variants due to a relative enrichment of low frequeny LOF variants (Figure S5a and main text). Purifying selection acts to remove common deleterious variants, thus causing a relative increase in low frequency LOF variants.


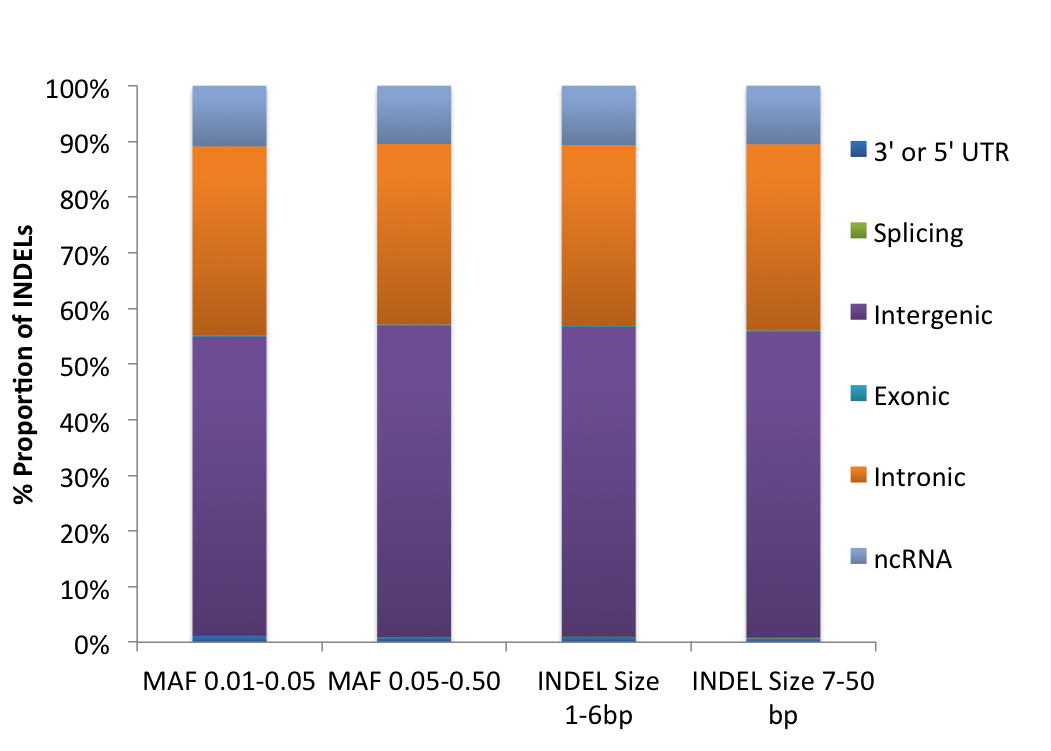
F**igure S5a: INDEL annotation with ANNOVAR**. CEU INDELs segmented by MAF and INDEL size was annotated with gene based filtering. There was a relative increase in exonic INDELs amongst low frequency vs. common INDELs. Large INDELs disproportionately concentrated amongst exonic RNA regions. YRI and CHB+JPT populations had similar distributions.

We see further evidence of purifying selection amongst INDELs > 6 bp. Using the following analysis (Figure S5b) INDELs > 6 bp were found to be more prevalent amongst exonic regions. The decrease in LOF variants amongst INDELs > 6 bp when compared to INDELs ≤ 6 bp, is likely due to purifying selection removing larger deleterious INDELs.


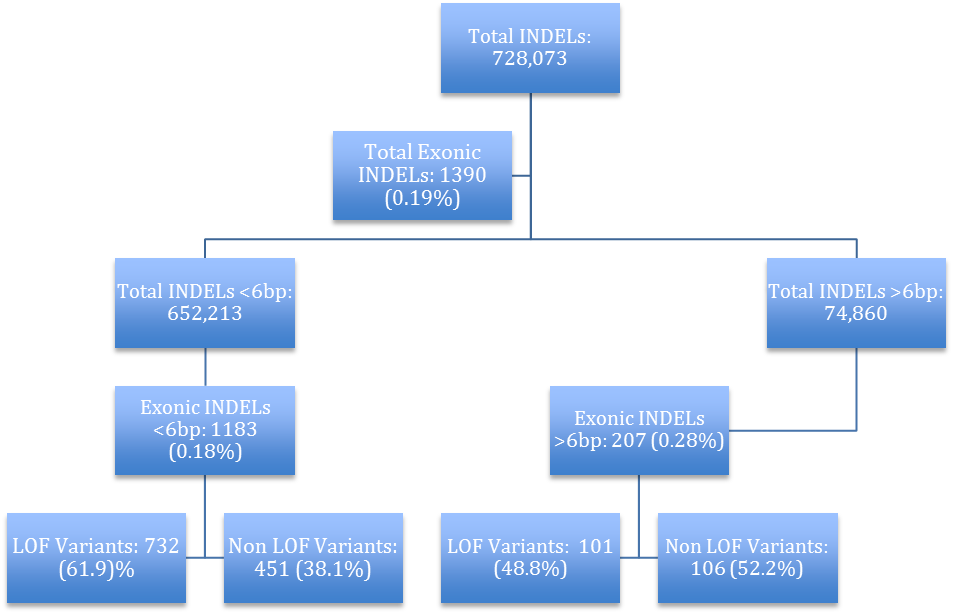


**Figure S5b: Comparison of INDELs ≤ 6 bp and INDELs >6 bp.** In this analysis INDELs ≤ 6 bp show higher prevalence of LOF variants than INDELs >6 bp, suggesting purifying selection.

In exploring the LD characteristics of LOF and non-LOF variants, we found that LD between the two was similar. LD for LOF INDELs was only marginally lower (Figure S6a) due to the large amount of common LOF variants that are well tagged by nearby SNPs (Figure S6b). In aggregate, tagging of LOF variants was reduced 1-6% relative to non- LOF variants depending on population. Further, as expected, LOF variants form longer haplotypes than non-LOF variants (Table S6).


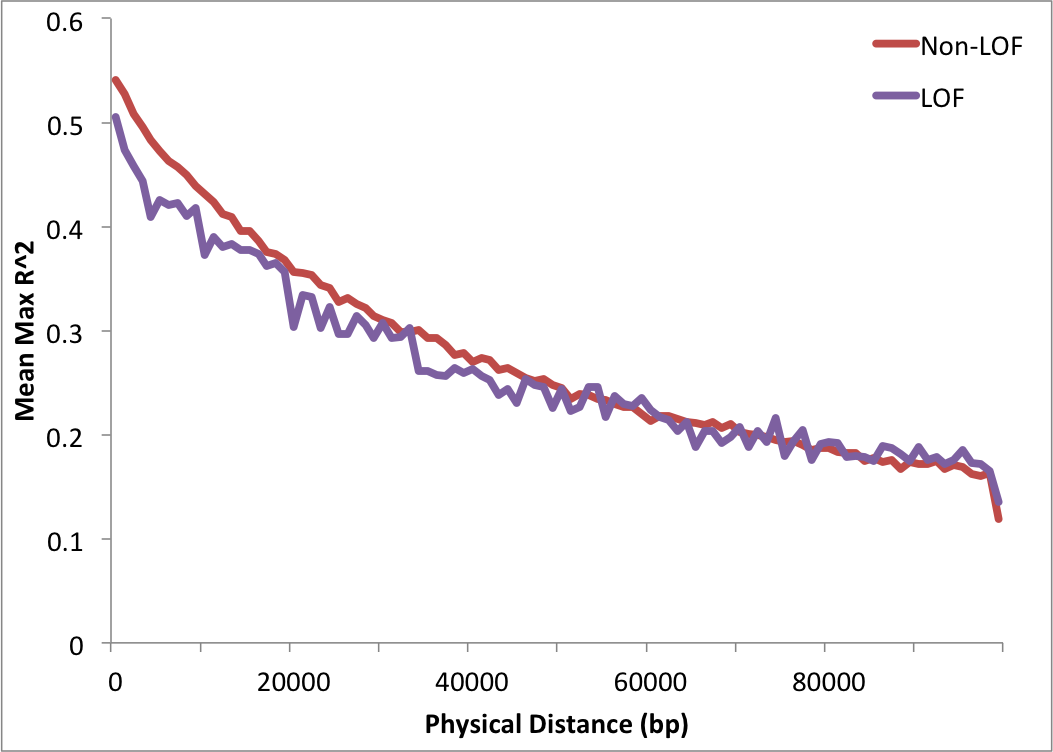


**Figure S6a: Plot of mean max r^2^ for LOF and non LOF CEU with 1000G SNPs.** LOF variants have lower LD due to their relative enrichment in rare and low frequency variants. At distances less than 14 kbp, LD for non-LOF variants is greater than LOF variants (Mann-Whitney, p<0.05).


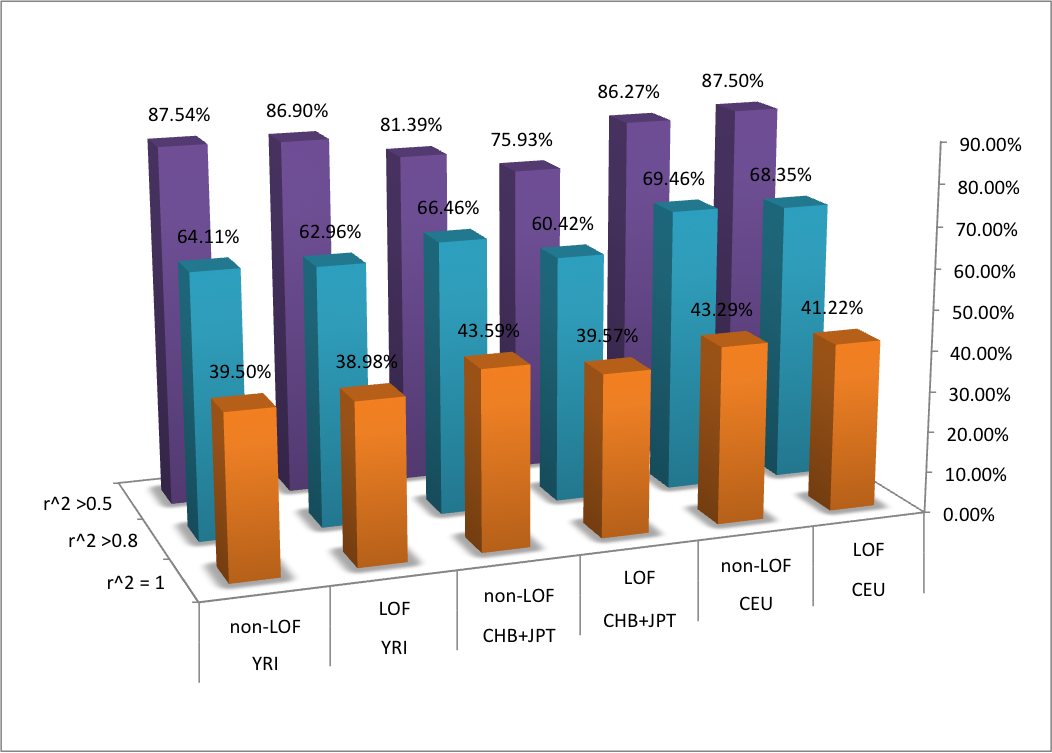


**Figure S6b: Percentage of LOF and non-LOF INDELs with at least one pairwise r^2^ value ≥ 0.5 (medium LD), ≥ 0.8 (high LD) or equal to 1 (perfect LD) when using 1000G SNPs.** Our results suggest that LOF and non-LOF variants are similarly tagged, with non-LOF variants tagged at slightly lower percentages.

| Population | LOF | 1^st^ Quartile ( bp) | Median ( bp) | 3rd Quartile. ( bp) |
| --- | --- | --- | --- | --- |
| CEU | LOF | 6882 | 19610 | 48580 |
| CEU | Not LOF | 5985 | 17010 | 39130 |
| CHBJPT | LOF | 6206 | 18010 | 42380 |
| CHBJPT | Not LOF | 5681 | 16050 | 37900 |
| YRI | LOF | 5266 | 15160 | 36530 |
| YRI | Not LOF | 4086 | 12850 | 33190 |

**Table S6: Haplotype length of LOF and non LOF INDELs to nearby SNPs that have high LD (r^2^ >0.8).** Due to the enrichment of low frequency INDELs amongst LOF variants, there is a increase in median length amongst LOF variants vs. non-LOF variants.

# 5) Imputation performance with SNPTools was more accurate than Impute2

In our study we relied upon two imputation engines to assess INDEL imputation power from SNPs. In comparing their overall imputation performance, we find that both engines can accurately impute common INDELs. However SNPTools and Impute2 vary in ref/alt and alt/alt imputation power. For common INDELs, IMPUTE2 imputed both ref/alt and alt/alt with an 80% concordance rate; however SNPTools had a concordance rate greater than 95%. For low frequency INDELs, SNPTools imputed ref/alt with an approximately 50% concordance rate versus 22% when using IMPUTE2. Both imputation engines could not impute low frequency homozygous alternative alleles. Both had imputation power of ~10% (Table S7a and Table S8a and Table S9a).

| **Imputation Engine** | **INDEL AF** | **ref/ref** | **ref/alt** | **alt/alt** | **Overall** |
| --- | --- | --- | --- | --- | --- |
| SNPTools | 1%-5% | 99.97% | 48.98% | 11.94% | 96.98% |
| Impute2 | 1%-5% | 98.99% | 21.84% | 10.62% | 94.53% |
| SNPTools | >5% | 99.65% | 97.61% | 96.45% | 98.75% |
| Impute2 | >5% | 94.09% | 80.33% | 78.41% | 88.38% |

**Table S7a: Comparison of imputation concordance for IMPUTE2 and SNPTOOLS**. SNPTools has better ref/alt performance and marginally better alt/alt performance. All populations were averaged together, with 1000G SNPs. Concordance is defined as the percentage of alleles that are accurately imputed as defined by the INDEL call set.

Both IMPUTE2 and SNPTools accurately impute LOF variants. However, SNPTools had better power, with > 95% of alleles accurately imputed (versus ~75-80% for IMPUTE2). Further the loss in accuracy when imputing non-LOF vs. LOF variants was greater in IMPUTE2 relative to SNPTools; power was reduced 2-4%.

| **Population** | **Panel** | **LOF** | **ref/ref** | **ref/alt** | **alt/alt** | **Overall** |
| --- | --- | --- | --- | --- | --- | --- |
| SNPTools | 1000G | y | 99.78% | 94.77% | 94.15% | 98.46% |
| Impute2 | 1000G | y | 96.38% | 75.25% | 75.71% | 90.78% |
| SNPTools | 1000G | n | 99.75% | 96.44% | 95.99% | 98.62% |
| Impute2 | 1000G | n | 95.69% | 78.81% | 79.06% | 89.98% |

**Table S7b: Comparison of Imputation performance of IMPUTE2 and SNPTOOLS for LOF INDELs**. All populations were averaged together, with 1000G SNPs. SNPTools has about 15-20% greater concordance than IMPUTE2 for ref/alt and alt/alt.

| **Population** | **Panel** | **MAF** | **ref/ref** | **ref/alt** | **alt/alt** | **Overall** |
| --- | --- | --- | --- | --- | --- | --- |
| CEU | 1000G | 1%-5% | 99.96% | 52.17% | 9.09% | 97.15% |
| CEU | 1000G | >5% | 99.53% | 98.19% | 97.12% | 98.90% |
| CEU | OMNI | 1%-5% | 100.00% | 58.86% | 13.89% | 97.61% |
| CEU | OMNI | >5% | 99.94% | 98.97% | 97.98% | 99.45% |
| CEU | HapMap | 1%-5% | 99.99% | 62.46% | 13.04% | 97.78% |
| CEU | HapMap | >5% | 99.87% | 99.02% | 98.11% | 99.45% |
| CHBJPT | 1000G | 1%-5% | 99.97% | 44.47% | 14.47% | 96.71% |
| CHBJPT | 1000G | >5% | 99.64% | 97.48% | 96.73% | 98.68% |
| CHBJPT | OMNI | 1%-5% | 99.78% | 54.98% | 7.50% | 97.11% |
| CHBJPT | OMNI | >5% | 99.94% | 99.19% | 97.86% | 99.51% |
| CHBJPT | HapMap | 1%-5% | 99.99% | 59.43% | 15.00% | 97.63% |
| CHBJPT | HapMap | >5% | 99.92% | 99.10% | 98.22% | 99.51% |
| YRI | 1000G | 1%-5% | 99.99% | 50.31% | 11.86% | 97.08% |
| YRI | 1000G | >5% | 99.76% | 97.13% | 95.27% | 98.67% |
| YRI | OMNI | 1%-5% | 100.00% | 54.77% | 11.29% | 97.35% |
| YRI | OMNI | >5% | 99.97% | 98.42% | 96.70% | 99.25% |
| YRI | HapMap | 1%-5% | 100.00% | 54.12% | 8.45% | 97.32% |
| YRI | HapMap | >5% | 99.93% | 98.24% | 96.18% | 99.16% |

**Table S8a: SNPTool imputation concordance for low frequency and common INDELs imputed with 1000G, OMNI and HapMap panels for each population:** Imputation performance mimics tagSNP data presented in Table S4. CEU and CHB+JPT show similar performance. YRI shows improved performance for low frequency INDELs relative to the other populations. Ref/Alt and Alt/alt show imputation performance of ~ 50% and ~10% for all populations. Imputation using OMNI and HapMap is > 98% for common INDELs, but ~40% for low frequency INDELs.

| **Population** | **Panel** | **LOF** | **ref/ref** | **ref/alt** | **alt/alt** | **Overall** |
| --- | --- | --- | --- | --- | --- | --- |
| CEU | 1000G | y | 99.62% | 95.36% | 95.16% | 98.41% |
| CEU | 1000G | n | 99.49% | 95.95% | 96.06% | 98.36% |
| CEU | OMNI | y | 99.62% | 95.64% | 96.21% | 98.55% |
| CEU | OMNI | n | 99.53% | 97.32% | 97.14% | 98.76% |
| CEU | HapMap | y | 99.65% | 96.87% | 96.35% | 98.86% |
| CEU | HapMap | n | 99.63% | 97.39% | 97.80% | 98.89% |
| CHBJPT | 1000G | y | 99.51% | 94.27% | 95.25% | 98.09% |
| CHBJPT | 1000G | n | 99.43% | 95.91% | 96.02% | 98.22% |
| CHBJPT | OMNI | y | 99.66% | 95.69% | 96.42% | 98.60% |
| CHBJPT | OMNI | n | 99.61% | 97.00% | 96.63% | 98.70% |
| CHBJPT | HapMap | y | 99.72% | 96.66% | 96.40% | 98.84% |
| CHBJPT | HapMap | n | 99.61% | 97.60% | 97.68% | 98.92% |
| YRI | 1000G | y | 99.69% | 93.10% | 90.61% | 97.96% |
| YRI | 1000G | n | 99.74% | 94.49% | 94.55% | 98.12% |
| YRI | OMNI | y | 99.75% | 94.51% | 92.93% | 98.44% |
| YRI | OMNI | n | 99.77% | 95.80% | 95.22% | 98.52% |
| YRI | HapMap | y | 99.75% | 94.63% | 95.19% | 98.55% |
| YRI | HapMap | n | 99.78% | 96.04% | 94.88% | 98.58% |

**Table S8b: SNPTool imputation concordance for LOF and non-LOF INDELs imputed with 1000G, OMNI and HapMap panels for each population.** Imputation performance is similar between LOF and non-LOF data.

We also compare the imputation of low frequency INDELs and low frequency SNPs from the 1000 Genome panels. We found that low frequency heterozygous and homozygous alternative SNPs are imputed with less accuracy than INDELs. We attribute this difference to the limitations of the INDEL dataset. Given the stringent filtering requirements of the Pilot project to reduce false positive discovery, the INDEL dataset is underrepresented for polymorphic and multi-allelic INDELs. This may cause inflation of the imputation concordance for INDELs.

| **Population** | **INDEL /SNP** | **ref/ref** | **ref/alt** | **alt/alt** | **Overall** |
| --- | --- | --- | --- | --- | --- |
| CEU | INDEL | 99.96% | 52.17% | 9.09% | 97.15% |
| CHB+JPT | INDEL | 99.97% | 44.47% | 14.47% | 96.71% |
| YRI | INDEL | 99.99% | 50.31% | 11.86% | 97.08% |
| CEU | SNP | 99.99% | 35.83% | 1.07% | 97.32% |
| CHB+JPT | SNP | 99.99% | 35.46% | 5.24% | 97.42% |
| YRI | SNP | 99.99% | 38.51% | 9.16% | 97.10% |

**Table S8c: Comparison of imputation concordance for low frequency INDELs and low frequency SNPs using SNPTOOLS**. We note that SNPTools has significantly less accuracy in ref/alt and alt/alt SNPs than INDELs. Also YRI imputed with higher accuracy, due to the higher relative proportion of low frequency SNPs in that population.

| **Population** | **Panel** | **MAF** | **ref/ref** | **ref/alt** | **alt/alt** | **Overall** |
| --- | --- | --- | --- | --- | --- | --- |
| CEU | 1000G | 1%-5% | 99.04% | 20.44% | 9.52% | 94.46% |
| CEU | 1000G | >5% | 94.45% | 82.32% | 78.74% | 89.07% |
| CHBJPT | 1000G | 1%-5% | 99.06% | 22.68% | 16.67% | 94.61% |
| CHBJPT | 1000G | >5% | 94.18% | 81.68% | 81.15% | 88.93% |
| YRI | 1000G | 1%-5% | 98.87% | 22.46% | 3.45% | 94.53% |
| YRI | 1000G | >5% | 93.71% | 76.80% | 74.31% | 87.17% |

**Table S9a: IMPUTE2 imputation concordance for low frequency and common INDELs imputed with 1000G for each population.**  We find imputation performance > 75% for all common INDELs (ref/alt and alt/alt) but dramatically worse performance for low frequency INDELs.

| **Population** | **Panel** | **LOF** | **ref/ref** | **ref/alt** | **alt/alt** | **Overall** |
| --- | --- | --- | --- | --- | --- | --- |
| CEU | 1000G | y | 96.60% | 77.38% | 75.80% | 91.23% |
| CEU | 1000G | n | 95.49% | 80.16% | 80.38% | 90.03% |
| CEU | HapMap | y | 96.98% | 81.65% | 84.76% | 92.80% |
| CEU | HapMap | n | 96.57% | 85.29% | 86.34% | 92.80% |
| CEU | OMNI | y | 97.22% | 81.31% | 81.97% | 92.93% |
| CEU | OMNI | n | 96.48% | 84.78% | 85.14% | 92.52% |
| CHBJPT | 1000G | y | 96.60% | 77.10% | 78.85% | 91.31% |
| CHBJPT | 1000G | n | 95.74% | 80.52% | 80.53% | 90.43% |
| CHBJPT | HapMap | y | 97.29% | 80.86% | 83.87% | 92.80% |
| CHBJPT | HapMap | n | 96.31% | 83.91% | 84.73% | 92.03% |
| CHBJPT | OMNI | y | 96.70% | 78.67% | 83.60% | 92.02% |
| CHBJPT | OMNI | n | 96.44% | 82.32% | 84.28% | 91.63% |
| YRI | 1000G | y | 95.92% | 70.54% | 71.05% | 89.72% |
| YRI | 1000G | n | 95.85% | 74.22% | 74.27% | 89.29% |
| YRI | HapMap | y | 96.58% | 77.12% | 83.23% | 92.12% |
| YRI | HapMap | n | 96.12% | 81.54% | 83.82% | 91.75% |
| YRI | OMNI | y | 95.89% | 77.50% | 79.29% | 91.47% |
| YRI | OMNI | n | 96.26% | 79.78% | 80.47% | 91.37% |

**Table S9b: IMPUTE2 imputation concordance for LOF and non-LOF INDELs imputed with 1000G, OMNI and HapMap panels for each population.** Imputation performance is marginally worse for LOF variants for all populations using 1000G SNPs.

## 6) INDELs ≤ 6 bp are imputed with marginally higher accuracy than INDELs >6 bp

After normalizing INDELs by MAF, we found that there was a small difference in imputation performance between INDELs ≤ 6 bp and INDELs >6 bp. Imputation for common INDELs was uniformly high with ref/alt and alt/alt concordance equal to 98.9% and 98.8% and 98.6% and 97.8% respectively for INDELs ≤ 6 bp and INDELs >6 bp. However, heterozygous variants for low frequency INDELs ≤ 6 bp were imputed at a slightly higher rate (53.2%) when compared to INDELs >6 bp (51.1%, p=0.036, 2-proportion test) (Figure 3b). This is likely due to the greater tagging in INDELs ≤ 6 bp, as discussed earlier. There was no difference in alt/alt imputation (p=0.35, 2-proportion test). (Figure S7)


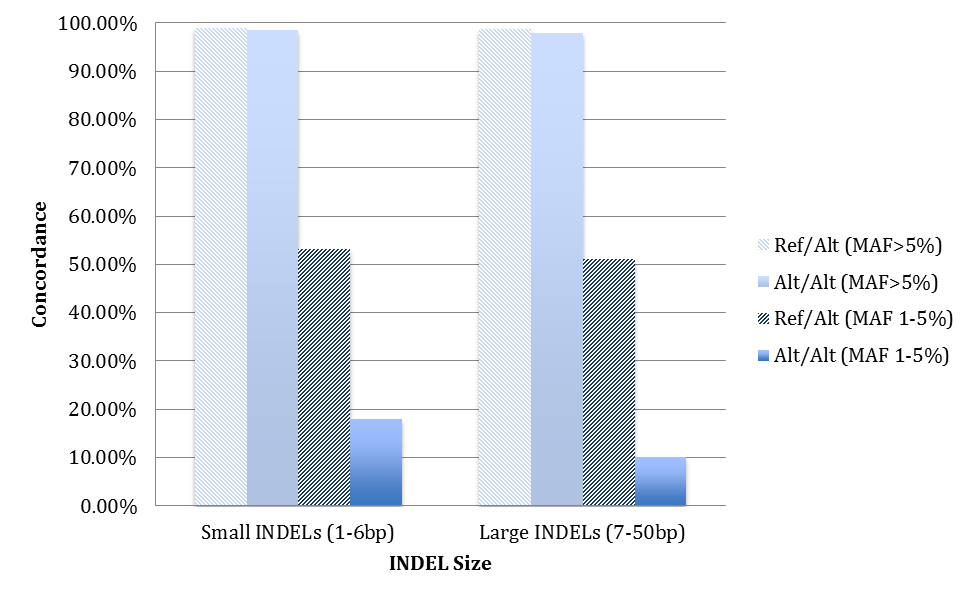


**Figure S7: Imputation performance for low frequency and common INDELs ≤ 6 bp and >6 bp**: While small, there was a significant difference in imputation performance of heterozygous alleles for low frequency INDELs ≤ 6 bp when compared INDELs >6 bp when using 1000G SNPs. There were no other statistically significant comparisons

1. Wang K, Li M, Hakonarson H: ANNOVAR: functional annotation of genetic variants from high-throughput sequencing data. *Nucleic Acids Res* 2010, 38:e16410.1093/nar/gkq603Available: Accessed 17 May 2011.

2. Kimura M: Rare variant alleles in the light of the neutral theory. *Mol. Biol. Evol* 1983, 1:84-93Available: Accessed 1 June 2011.

3. A second generation human haplotype map of over 3.1 million SNPs: *Nature* 2007, 449:851-86110.1038/nature06258Available: Accessed 28 March 2011.

4. A haplotype map of the human genome: *Nature* 2005, 437:1299-132010.1038/nature04226Available: Accessed 1 May 2011.
